# RFX1 regulates CD70 and CD11a expression in lupus T cells by recruiting the histone methyltransferase SUV39H1

**DOI:** 10.1186/ar3214

**Published:** 2010-12-30

**Authors:** Ming Zhao, Xiaoyan Wu, Qing Zhang, Shuangyan Luo, Gongping Liang, Yuwen Su, Yixin Tan, Qianjin Lu

**Affiliations:** 1 Department of Dermatology, Second Xiangya Hospital, Central South University, Hunan Key Laboratory of Medical Epigenomics, No. 139 Renmin Middle Rd, Changsha, Hunan 410011, PR China

## Abstract

**Introduction:**

Regulatory factor X-box 1 (RFX1) can interact with DNA methyltransferase 1 (DNMT1) and histone deacetylase 1 (HDAC1), and RFX1 down-regulation contributes to DNA hypomethylation and histone H3 hyperacetylation at the cluster of differentiation (CD) 11a and CD70 promoters in CD4^+ ^T cells of patients with systemic lupus erythematosus (SLE). This leads to CD11a and CD70 overexpression, thereby triggering autoimmune responses. In order to provide more insight into the epigenetic mechanisms leading to the deregulation of autoimmune-related genes in SLE, we asked whether RFX1 is involved in regulating histone 3 lysine 9 (H3K9) tri-methylation at the CD11a and CD70 promoters in SLE CD4^+ ^T cells.

**Methods:**

CD4^+ ^T cell samples were isolated from 15 SLE patients and 15 healthy controls. H3K9 tri-methylation levels were measured by chromatin immunoprecipitation (ChIP) and real-time quantitative PCR. CD4^+ ^T cells were transfected with plasmids using the Human T cell Nucleofector Kit. RFX1 and histone methyltransferase suppressor of variegation 3-9 (Drosophila) homolog 1 (SUV39H1) interaction was determined by co-immunoprecipation (co-IP) and Western blot and immunofluorescence staining. CD11a and CD70 mRNA levels were measured by real-time RT-PCR.

**Results:**

H3K9 tri-methylation levels were significantly reduced within the CD11a and CD70 promoter regions in SLE CD4^+ ^T cells. RFX1 co-immunoprecipitated with SUV39H1 at the CD11a and CD70 promoters in healthy control CD4^+ ^T cells. Overexpressing or knocking-down RFX1 revealed that RFX1 expression correlated with H3K9 tri-methylation levels, as well as CD11a and CD70 expression levels in CD4^+ ^T cells.

**Conclusions:**

RFX1 recruits SUV39H1 to the promoter regions of the CD11a and CD70 genes in CD4^+ ^T cells, thereby regulating local H3K9 tri-methylation levels. These findings shed further light on the central role of RFX1 down-regulation in the epigenetic de-repression of auto-immune genes in SLE.

## Introduction

Systemic lupus erythematosus (SLE) is a chronic autoimmune disease characterized by excess production of autoantibodies. Multiple studies have demonstrated the important role of epigenetic alterations in triggering the hyper-activation of T lymphocytes that leads to lupus and lupus-like diseases [1-3]. T-cell autoreactivity in lupus is thought to be due in part to the overexpression of adhesion molecule lymphocyte function-associated antigen 1 (LFA-1, composed of cluster of differentiation (CD) 11a and CD18 subunits) [4,5], and of CD70 (TNFSF7), which induces B cells to over-produce autoantibodies [6,7]. Our previous studies have confirmed that DNA hypomethylation and histone hyperacetylation of CD11a and CD70 promoter regions contribute to their overexpression in SLE CD4^+ ^T cells [8-10]. However, the mechanisms leading to deregulated epigenetic modifications at the CD11a and CD70 gene loci are not completely understood.

The transcription factor regulatory factor X-box 1 (RFX1), the first cloned member of the RFX family, is down-regulated in CD4^+ ^T cells of SLE patients [11]. RFX1 contains a C-terminal repressive region, an overlapping dimerization domain, and an N-terminal activation domain, and is capable of both activating and repressing target gene transcription[0] depending on the cellular context [12]. Although the mechanisms by which RFX1 exerts its activation or repression activity have only been partially elucidated, it is known that RFX1 behaves as a potent transcriptional repressor in CD4^+ ^T cells [10]. In these cells, RFX1 binds to target genes, including CD11a and CD70, and recruits the transcriptional co-repressors histone deacetylase 1 (HDAC1) and DNA methyltransferase1 (DNMT1). This leads to local histone hypoacetylation and DNA hypermethylation and consequently to the suppression of target gene expression [10]. In a previous study, we found that RFX1 is significantly down-regulated in SLE CD4^+ ^T cells. We also demonstrated that RFX1 forms a stable complex with HDAC1 and DNMT1 in the nucleus of CD4^+ ^T cells, and that down-regulating RFX1 in these cells increases histone acetylation and decreases DNA methylation at the CD11a and CD70 promoter regions, epigenetic changes that lead to the de-repression of CD11a and CD70 [10].

Another epigenetic mechanism for repressing gene transcription is the methylation of specific lysine residues in histones, a modification that is critical to shaping repressive chromatin structures [13,14]. Among the histone methyltransferases involved in this process, the best characterized is the suppressor of variegation 3-9 (Drosophila) homolog 1 (SUV39H1). By tri-methylating lysine 9 in histone H3 (H3K9) SUV39H1 is able to generate a binding site for the transcriptional repressor heterochromatin-associated protein 1 (HP1) [15,16]. Therefore, H3K9 tri-methylation is involved in gene repression and serves as a marker for the establishment of a stable heterochromatin configuration [17]. In the present study, we show that RFX1 binds to CD11a and CD70 promoter DNA in CD4^+ ^T cells where it recruits SUV39H1 and regulates H3K9 tri-methylation levels. We further reveal that the down-regulation of RFX1 in SLE CD4^+ ^T cells reduces local H3K9 tri-methylation levels around the promoters of CD11a and CD70, thus further contributing to the de-repression of these critical auto-immune factors.

## Materials and methods

### Patients and controls

Patient demographics and treatment regimens are shown in Table 1. SLE patients (mean age 27 ± 6 yrs) were recruited from outpatient clinics of the Second Xiangya Hospital Central South University. All patients fulfilled at least four of the SLE classification criteria of the American College of Rheumatology [18]. Lupus disease activity was assessed using the SLE Disease Activity Index (SLEDAI) [19]. Healthy controls (mean age 25 ± 3 yrs) were recruited from medical staff at the Second Xiangya Hospital. This study was approved by the human ethics committee of the Central South University Xiangya Medical School, and written informed consent was obtained from all subjects. Patients and controls were age- and sex-matched in all experiments.

**Table 1:** Patient demographics and medications

Patient	SLEDAI score	Medications
1	14	None
2	14	None
3	14	Pred15mg/d
4	10	Pred 10 mg/d
5	14	None
6	14	None
7	19	Pred 20 mg/d
8	15	Pred 30 mg/d
9	14	Pred 10 mg/d
10	24	Pred 20 mg/d
11	16	Pred 5 mg/d
12	20	None
13	18	Pred 15 mg/d
14	20	Pred 2.5 mg/d
15	14	Tria 8 mg/d

Pred, Prednisone; SLEDAI, Systemic Lupus Erythematosus Disease Activity Index; Tria, Triamcinolone.

### Isolation, culturing and transfection of T cells

A total of 60 ml of venous peripheral blood was withdrawn from each patient and control subject and preserved with heparin. CD4^+ ^T cells were isolated by positive selection using CD4 beads, according to protocols provided by the manufacturer (Miltenyi, Bergisch Gladbach, Germany; purity was generally higher than 95%), and cultured in human T cell culture medium (Lonza, Walkersville, MD, USA). CD4^+ ^T cells were transfected with plasmids using the Human T cell Nucleofector Kit and Amaxa nucleofector (Lonza). In brief, CD4^+ ^T cells were harvested and resuspended in 100 μl human T cell nucleofector solution. The cell suspension was then mixed with 10 μg empty plasmids (pSUPER or pSG5) or plasmid vectors encoding an RFX1-targeting siRNA (pSuper.RFX1) or full-length RFX1 cDNA (pSG5-RFX1, both provided by Dr. Yosef Shaul, Weizmann Institute of Science, Rehovot, Israel). The mix was electrotransfected using the nucleofector program V-024 in the Amaxa nucleofector. Transfected cells were cultured in human T cell culture medium and harvested 48 hours later.

### Chromatin immunoprecipitation (ChIP)

ChIP analysis was performed according to the instructions provided with the ChIP assay kit (Millipore, Billerica, MA, USA). In brief, CD4^+ ^T cells were fixed for eight minutes at RT with 1% formaldehyde. Glycine was then added to a final concentration of 0.125 M to quench the formaldehyde. Cells were pelleted, washed once with ice-cold PBS, and lysed. Lysates were pelleted, resuspended, and sonicated to reduce DNA to 500 to 1,000 base pair fragments. Chromatin was precipitated with protein A agarose beads for one hour and then incubated with tri-methylated H3K9 antibody (Abcam, Cambridge, MA, USA) or control rabbit IgG (Millipore) overnight. The immunocomplexes were precipitated once again with protein A agarose beads, washed, and eluted in 100 ml of TE with 0.5% SDS and 200 mg/ml proteinase K. Precipitated DNA was further purified with phenol/chloroform extranction and ethanol before amplifying target DNA by reverse transcriptase-polymerase chain reaction (RT-PCR). Primers used were as follows: CD11a, 5'-CAGCCTGTTGCCTCTGTGAGA-3' (forward) and 5'-GGCAGCT CCTTGTTTACTCC-3' (reverse); and CD70, 5'-GGGCGTCTACTTGCTTCA-3' (forward) and 5'-CCTGCATCCTGGCAACTGC-3' (reverse).

### Real-time quantitative polymerase chain reaction (qPCR)

qPCR was used to quantify the abundance of DNA fragments of CD11a and CD70 promoter DNA fragments. These experiments were performed with 20 μl reaction volumes containing 10 μl 2×SYBR^® ^Premix Ex Taq (TaKaRa Biotech (Dalian) Co., Dalian, China), 0.4 μM of each primer, 1 μl of cDNA template, and 8.2 μl deionized water. PCR amplifications were done in a Rotor-Gene3000 (Corbett Research, Mortlake, NSW, Australia) using the following parameters: 95°C for 10 s, 40 cycles through 95°C for 5 s, 58°C to 60°C for 31 s. Melting curve analysis (from 65°C to 95°C, followed by cooling to 40°C) was also performed to exclude non-specific PCR products. All PCR products were checked by melting curve analysis to exclude the possibility of multiple products or incorrect product size. PCR analyses were conducted in triplicate for each sample.

### RNA isolation and real-time quantitative RT-PCR

Total RNA was isolated from CD4^+ ^T cells using the RNeasy mini kit (Qiagen, Valencia, CA, USA). Real-time quantitative RT-PCR was performed using a Rotor-Gene3000 (Corbett Research) and mRNA levels were quantified using the One Step PrimeScript RT-PCR Kit (TaKaRa Biotech (Dalian) Co). A dilution series of sample RNA was also included to generate a standard curve used to calculate relative concentrations of transcript in each RNA sample. β-actin was also amplified and used as a loading control. Primers used were as follows: CD11a, 5'-TGAGAGCAGGCTATTT GGGTTAC-3' (forward) and 5'-CGGCCCATGTGCTGGTAT-3' (reverse); CD70, 5'- CACACTCTGCACCTCACT-3' (forward) and 5'-CACCCACTGCACTCCAAAGA-3' (reverse); and β-actin, 5'-CGCGAGAAGATGACCCAGAT-3' (forward) and 5'-GCAC TGTGTTGGCGTACAGG-3' (reverse).

### Western blots

Western blots were performed as described previously [20]. Primary antibodies used included: anti-SUV39H1 (1:1000; Santa Cruz Biotechnology, CA, USA), anti-RFX1 (1:100; Santa Cruz Biotechnology) Blots were visualized using SuperSignal West Pico Chemiluminescent Substrate (Pierce, Rockford, IL, USA) and exposed to X-ray films. Band densities were quantified using Quantity One software (Bio-Rad, Hercules, CA, USA).

### Co-immunoprecipitation (co-IP)

Whole CD4^+ ^T cell lysates were obtained by resuspending CD4^+ ^T cell pellets in RIPA buffer. Lysates were incubated overnight with RFX1 antibody (Santa Cruz Biotechnology) before being absorbing with protein A/G PLUS-agarose beads (Millipore). Precipitated immunocomplexes were released by boiling with 2 × SDS electrophoresis sample buffer and prepared for western blot analysis.

### Immunofluorescence analysis

Fixed and permeabilized cells were incubated with anti-RFX1 (1:100; Santa Cruz Biotechnology), anti-SUV39H1 (1:200; Santa Cruz Biotechnology) and anti-Bcl-2 (1:100; Santa Cruz Biotechnology) antibodies, followed by FITC- and TRITC-conjugated secondary antibodies (Santa Cruz Biotechnology) using standard procedures. Nuclei were counter-stained with DAPI (Santa Cruz Biotechnology). Images were captured using a Zeiss LSM5 confocal microscope (Carl Zeiss, Thornwood, NY, USA).

### Statistical analysis

Results are expressed as mean ± SD. Data were analyzed by ANOVA followed by the unpaired Student's *t*-test for multiple comparisons. All analyses were preformed with SPSS 13.0 software (SPSS Inc., Chicago, IL, USA). Significance was set as *P *≤ 0.05.

## Results

### The H3K9 tri-methylation levels at the CD11a and CD70 promoters in SLE CD4^+ ^T cells

**Figure 1:**
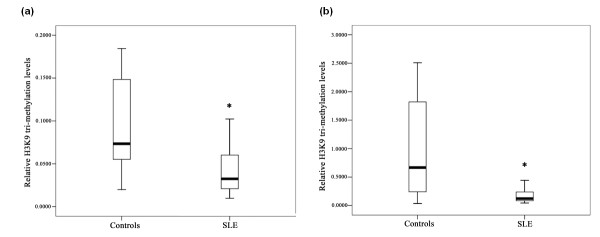
H3K9 tri-methylation levels in the CD11a **(a) **and CD70 **(b) **promoters regions of SLE and healthy control CD4^+ ^T cells detected by ChIP and PCR (*, *P *< 0.01; **, *P *< 0.05).

Our group previously showed that global H3K9 is globally hypomethylated in active and inactive lupus CD4^+ ^T cells [21]. To investigate the effect of aberrant histone methylation on the expression of the auto-immune related genes CD11a and CD70, we analyzed H3K9 tri-methylation levels at the CD11a and CD70 genomic loci in CD4^+ ^T cells from SLE patients and healthy controls (*n *= 15 per group). ChIP-qPCR analysis revealed that the H3K9 in the promoter regions of CD11a and CD70 are significantly hypomethylated in SLE CD4^+ ^T cells compared with control CD4^+ ^T cells (Figure 1).

### RFX1 recruits SUV39H1 to the CD11a and CD70 promoters in CD4^+ ^T cells

**Figure 2:**
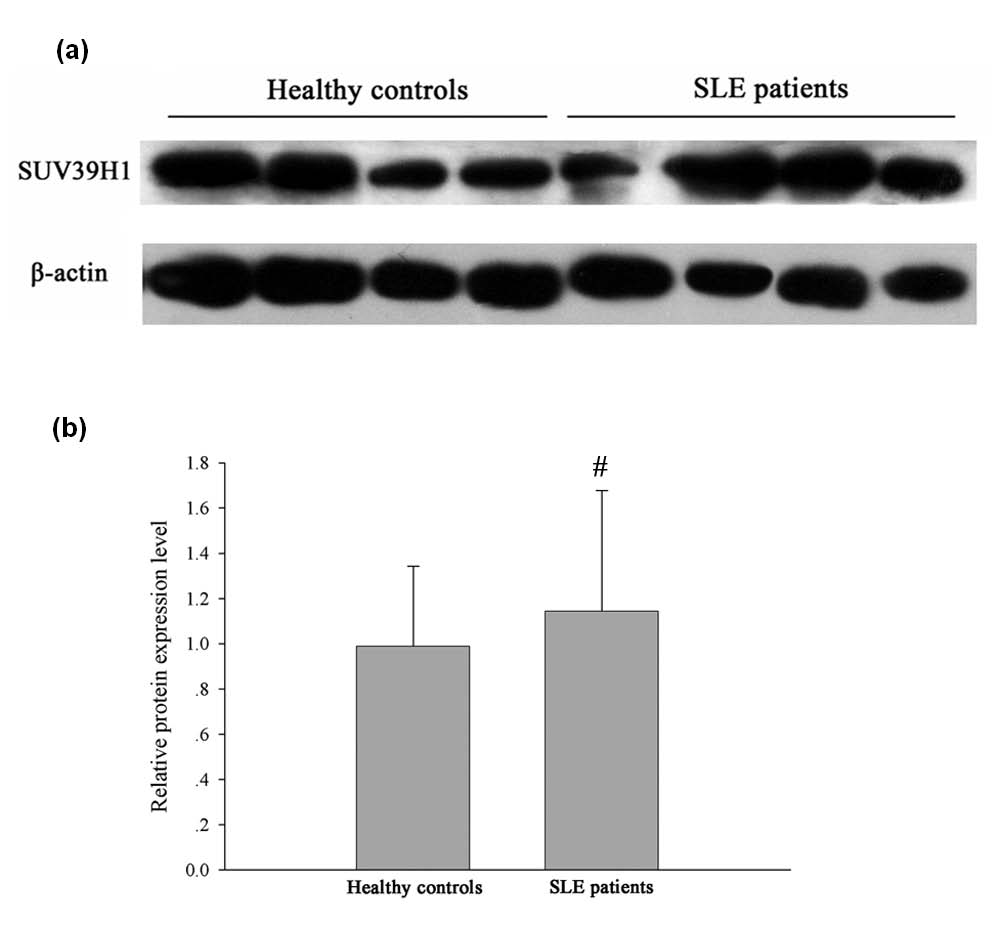
**SUV39H1 protein levels from SLE patients (*n *= 15) and healthy controls (*n *= 15) measured by Western blot**. **(a) **Representative blots of SUV39H1 and β-actin (loading control) in SLE patient and healthy control CD4^+ ^T cells (*n *= 4 per group). **(b) **Quantitative analysis of SUV39H1 band intensities normalized to β-actin (#, *P *> 0.05).

In mammals, H3K9 tri-methylation is primarily, if not exclusively, due to the action of SUV39H1 [22]. We, therefore, examined SUV39H1 expression in SLE CD4^+ ^T cells. As shown in Figure 2, we found that SUV39H1 protein levels do not differ significantly between CD4^+ ^T cells from SLE patients and healthy controls.

**Figure 3:**
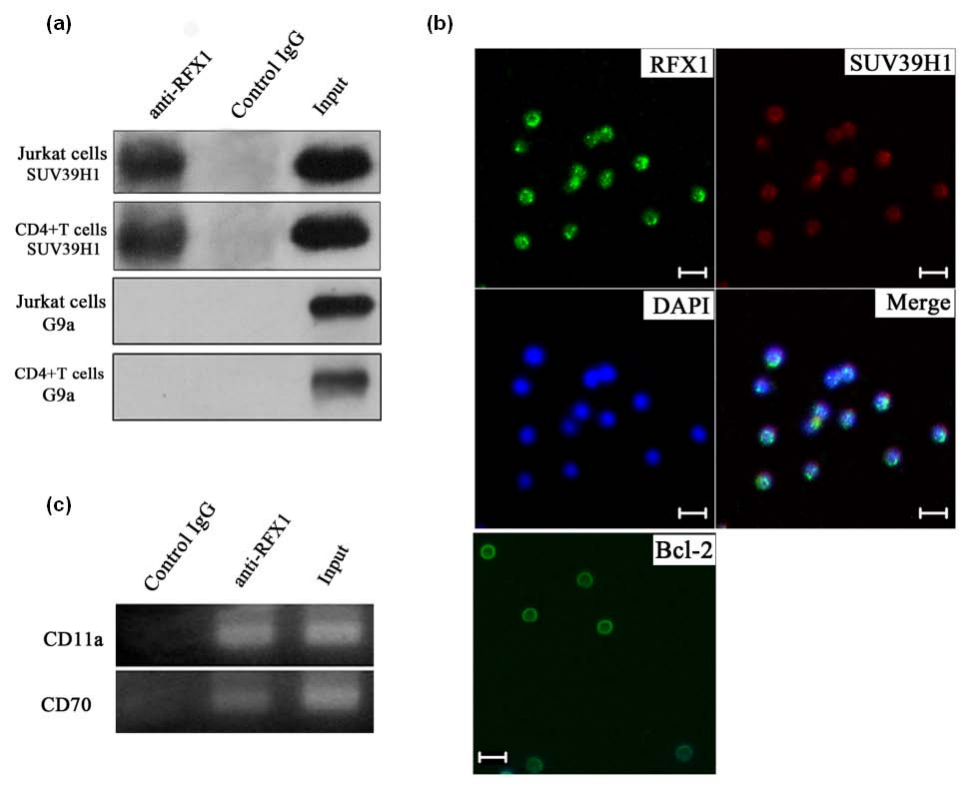
**SUV39H1 physically interacts with RFX1, and is recruited to CD11a and CD70 promoters**. **(a) **Anti-SUV39H1 western blots of Jurkat cells (top) and healthy CD4^+ ^T cells (bottom) lysates following immunoprecipitation with the antibodies indicated at the top. **(b) **CD4^+ ^T cells from healthy controls co-immunolabeled with anti-RFX1 (green) and anti-SUV39H1 (red). DAPI (blue) was used to label cell nuclei. Merged images are also shown. Anti-Bcl-2 antibody was included as control to cytoplasm of CD4^+ ^T cells (green). Scale bar, 10 μm. **(c) **Anti-SUV39H1 ChIP assay of CD4^+ ^T cell lysates. CD11a and CD70 promoters were identified by PCR amplification of the DNA fragments precipitated with SUV39H1 antibody.

We have previously demonstrated that the transcription factor RFX1 recruits the chromatin-modifying enzymes HDAC1 and DNMT1 to the promoter regions of CD11a and CD70 in CD4^+ ^T cells [10]. To determine whether RFX1 can associate with SUV39H1 and regulate H3K9 tri-methylation at CD11a and CD70 promoters, we performed co-IP and Western blot analyses of CD4^+ ^T cell lysates. SUV39H1 and RFX1 were found to physically interact in lysates from healthy subjects (Figure 3a). In contrast, G9a, another histone methyltransferase, did not co-IP with RFX1 (Figure 3a), suggesting that RFX1 binds specifically to SUV39H1. Furthermore, co-immunolabeling confirmed that SUV39H1 and RFX1 proteins co-localize in the nucleus of CD4^+ ^T cells (Figure 3b). We then used a ChIP assay to determine whether SUV39H1 was also recruited to CD11a and CD70 promoter regions. Figure 3c shows that SUV39H1 can bind to DNA fragments of the endogenous CD11a and CD70 promoter regions from healthy control CD4^+ ^T cells.

### RFX1 down-regulation causes overexpression of CD11a and CD70 by reducing promoter H3K9 tri-methylation levels

**Figure 4:**
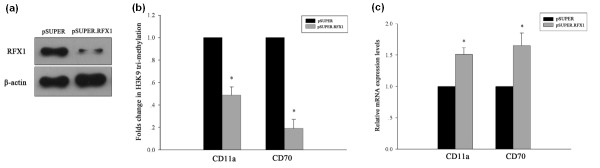
**CD4**^**+ **^**T cells after transfection with the RFX1-siRNA expression vector pSUPER.RFX1 or pSUPER (negative control)**. **(a) **Anti-RFX1 western blot of lysates from transfected CD4^+ ^T cells. Anti-β-actin Western blot is included as a loading control. **(b) **H3K9 tri-methylation levels at the CD11a and CD70 promoters in pSUPER.RFX1-transfected CD4^+ ^T cells relative to pSUPER-transfected cells (*, *P *< 0.01). C: CD11a and CD70 mRNA levels in pSUPER.RFX1-transfected CD4^+ ^T cells relative to pSUPER-transfected cells (*, *P *< 0.01). Data represent the mean ± SD of three independent experiments per group.

To assess whether RFX1 is involved in regulating H3K9 tri-methylation levels at the CD11a and CD70 promoter loci, we knocked-down RFX1 expression in healthy CD4^+ ^T cells by transfecting with an RFX1-siRNA expression vector, pSUPER.RFX1. Compared with cells transfected with empty pSUPER vector (negative control), RFX1 protein levels were decreased by approximately 90% 48 hours after pSUPER.RFX1 transfection (Figure 4a). We then used qPCR to measure the levels of CD11a and CD70 promoter DNA fragments after immunoprecipitating histone-DNA complexes with anti-tri-methylated H3K9 antibody. Significantly less CD11a and CD70 promoter DNA was amplified in pSUPER.RFX1-transfected CD4^+ ^T cells compared with negative controls, indicating a significant reduction in H3K9 tri-methylation levels (Figure 4b). In addition, CD11a and CD70 mRNA expression was significantly up-regulated compared with negative controls (Figure 4c).

### RFX1 overexpression up-regulates H3K9 tri-methylation at the CD11a and CD70 promoter loci

**Figure 5:**
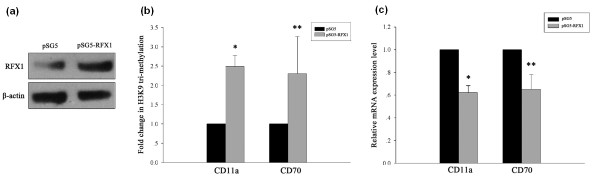
**SLE CD4**^**+ **^**T cells after transfection with RFX1 expression vector pSG5-RFX1, or with pSG5 (negative control)**. **(a) **Anti-RFX1 western blot of lysates from transfected CD4^+ ^T cells. Anti-β-actin western blot is included as a loading control. **(b) **H3K9 tri-methylation levels at the CD11a and CD70 promoters in pSG5-RFX1-transfected CD4^+ ^T cells relative to pSG5-transfected cells (*, *P *< 0.01; **, *P *< 0.05). **(c) **CD11a and CD70 mRNA levels in pSG5-RFX1-transfected CD4^+ ^T cells relative to pSG5-transfected cells (*, *P *< 0.01; **, *P *< 0.05). Data represent the mean ± SD of three independent experiments per group.

We have previously shown that RFX1 overexpression can increase DNA methylation and decrease histone acetylation levels at the CD11a and CD70 promoters, leading to reduced CD11a and CD70 expression in SLE CD4^+ ^T cells [10]. In this study, we asked whether overexpressing RFX1 could restore the aberrant histone tri-methylation status of SLE CD4^+ ^T cells. Figure 5a shows a significant increase in RFX1 protein levels in SLE CD4^+ ^T cells transfected with the RFX1 expression vector pSG5-RFX1. CD11a and CD70 promoter H3K9 tri-methylation levels were significantly higher in RFX1-overexpressing SLE CD4^+ ^T cells compared to control-transfected SLE CD4^+ ^T cells (Figure 5b), and this change correlated with a decrease in the expression of CD11a and CD70 mRNA (Figure 5c).

## Discussion

CD11a and CD70 are both overexpressed in CD4^+ ^T cells of lupus patients, and the degree of overexpression is directly proportional to disease activity [7]. CD11a overexpression contributes to autoreactive responses [8], while CD70 overexpression leads to overstimulation of IgG synthesis in B cells [9]. The expression of both genes is also increased in T cells treated with the methylation inhibitor 5-azacitidine, suggesting that DNA methylation is involved in regulating the expression of these genes [7,8]. Our previous studies have confirmed that the promoters of CD11a and CD70 genes are hypomethylation in SLE CD4^+ ^T cells, and that this contributes to their overexpression [7-9,23]. Our group has evidence suggesting that histone acetylation levels at the CD11a and CD70 promoter loci are higher in SLE CD4^+ ^T cells than in healthy controls (Lu Q, unpublished data). These findings demonstrate that changes in epigenetic regulatory factors lead to the up-regulation of CD11a and CD70 expression in CD4^+ ^T cells of SLE patients.

H3K9 methylation is one of the most prevalent and stable histone modifications, and is involved in both gene repression and heterochromatin formation. In mammals, heterochromatic regions are highly tri-methylated on H3K9, whereas euchromatin regions are enriched with mono- and di-methylated H3K9 [24]. Previous reports have shown that H3K9 tri-methylation acts as an epigenetic marker of transcriptional suppression, and the disruption of normal H3K9 tri-methylation levels is linked to a number of diseases. Increased H3K9 tri-methylation levels are associated with the silencing of tumor suppressor genes such as *P16*, *P14*, *MLH1 *and *MGMT *in cancer cells [25,26]. In contrast, H3K9 tri-methylation levels are significantly decreased at the promoters of key inflammatory genes *IL-6*, *MCSF *and *MCP-1 *promoters in vascular smooth muscle cells of mice with a type 2 diabetes-like condition [27]. In the present study, we found that H3K9 tri-methylation levels within the CD11a and CD70 promoter regions were decreased in SLE CD4^+ ^T cells compared with healthy controls, consistent with the global H3K9 hypomethylation of T cells from SLE patients that we reported previously [21]. Together, these findings suggest that decreased H3K9 tri-methylation levels is one of the mechanisms by which CD11a and CD70 expression becomes up-regulated in SLE CD4^+ ^T cells.

Many H3K9-specific histone methyltransferases (HMTs) have been characterized, all of which contain a conserved Su(var)3-9, Enhancer-of-zeste, Trithorax (SET) domain [13]. The most well described H3K9 HMT are the tri-methylase Suv39H1 and 2/KMT1A and -1B, which contribute mainly to the establishment of pericentric heterochromatin [22,28]. Our results showed that levels of SUV39H1 protein were not significantly different between SLE CD4^+ ^T cells and healthy controls, consistent with the mRNA levels detected by real-time PCR reported in our previous study [21]. Interestingly, our ChIP experiments demonstrated that SUV39H1 could bind to the promoter region of CD11a and CD70, suggesting that decreased tri-methylation at these promoters could potentially be associated with a reduction of SUV39H1 activity within these chromosomal regions.

Studies have shown that SUV39H1 can be recruited by transcription factors to the promoter region of specific genes where it then regulates histone methylation levels locally, and thereby represses target gene expression [29,30]. In a previous study, we screened SLE CD4^+ ^T cells for differential transcription factor activity using a microarray-based technique. Among our results, we found that RFX1 is significantly down-regulated in SLE patient T cells and also demonstrated that reduced RFX1 expression leads to the de-repression of CD11a and CD70 in SLE CD4^+ ^T cells. This was found to be due to the reduction of HDAC1 and DNMT1 recruitment, which in turn leads to an increase of histone acetylation and a decrease in DNA methylation within CD11a and CD70 promoter regions [10]. In the present study we found that SUV39H1 could directly interact with RFX1 and the two molecules co-localized in the nucleus of CD4^+ ^T cells. Furthermore, we found that RFX1 levels directly correlated with H3K9 tri-methylation levels. Knocking-down RFX1 in healthy control CD4^+ ^T cells reduced the level of H3K9 tri-methylation at the CD11a and CD70 promoters, whereas overexpressing RFX1 in SLE CD4^+ ^T cells had the opposite effect. Thus, we infer that decreased H3K9 tri-methylation in SLE CD4^+ ^T cells is partly due to the reduction in RFX1 protein levels.

Taken together, our previous studies and the present findings suggest that RFX1 restricts the expression of CD11a and CD70 and possibly other autoimmune-related genes by maintaining a repressive chromatin state of their promoters. The down-regulation of RFX1 CD4^+ ^T cells in patients with SLE contributes to, and perhaps triggers, the decondensation of chromatin around the CD11a and CD70 gene loci by disrupting the normal regulation of epigenetic modifications in these regions. This leads to the CD11a and CD70 overexpression and the onset of T-cell auto-reactivity [10]. Our findings provide new evidence of the key role of transcription factors in regulating the chromatin status of their target genes.

## Conclusions

In summary, our data further support a model whereby the binding of RFX1 to specific regulatory regions in healthy CD4^+ ^T cells leads to the recruitment of SUV39H1, HDAC1 and DNMT1 core complexes, which together inhibit the expression of RFX1 target genes. However, in CD4^+ ^T cells of patients with SLE, the combined activity of these RFX1-dependent transcriptional repressor complexes is insufficient due to the down-regulation of RFX1, thus leading to the de-repression of auto-immune related target genes, such as CD11a and CD70.

## Abbreviations

CD: cluster of differentiation; ChIP: chromatin immunoprecipitation; co-IP: co-immunoprecipitation; DNMT1: DNA methyltransferase1; H3K9: lysine 9 of histone H3; HDAC1: histone deacetylase 1; HMTs: histone methyltransferases; LFA-1: lymphocyte function-associated antigen 1; qPCR: quantitive PCR; RFX1: regulatory factor X-box 1; RT-PCR: reverse transcriptase- polymerase chain reaction; SLE: systemic lupus erythematosus; SLEDAI: SLE Disease Activity Index; SUV39H1: suppressor of variegation 3-9 (Drosophila) homolog 1.

## Competing interests

The authors declare that they have no competing interests.

## Authors' contributions

MZ and XW contributed equally to this work. MZ and XW performed most of the experiments and data analysis. QL helped in the design of the study and the critical analysis of the data. MZ and QL wrote the manuscript. QZ, SL, and YS assisted in the recruitment of patients, isolation of CD4^+ ^T cells and ChIP analysis. GL provided technical assistance. All authors read and approved the manuscript.
